# The Effect of Recombinant Growth Hormone Treatment in Children with Idiopathic Short Stature and Low Insulin-Like Growth Factor-1 Levels

**DOI:** 10.4274/jcrpe.2111

**Published:** 2015-12-03

**Authors:** Zeynep Şıklar, Pınar Kocaay, Emine Çamtosun, Mehmet İsakoca, Bülent Hacıhamdioğlu, Şenay Savaş Erdeve, Merih Berberoğlu

**Affiliations:** 1 Ankara University Faculty of Medicine, Department of Pediatric Endocrinology, Ankara, Turkey

**Keywords:** final height, growth hormone treatment, Idiopathic short stature, insulin-like growth factor deficiency

## Abstract

**Objective:**

Idiopathic short stature (ISS) constitutes a heterogeneous group of short stature which is not associated with an endocrine or other identifiable cause. Some ISS patients may have varying degrees of insulin-like growth factor-1 (IGF-1) deficiency. Recombinant growth hormone (rGH) treatment has been used by some authors with variable results. Reports on long-term rGH treatment are limited.

**Methods:**

In this study, 21 slowly growing, non-GH-deficient ISS children who received rGH treatment for 3.62±0.92 years were evaluated at the end of a 5.42±1.67-year follow-up period. The study group included patients with low IGF-1 levels who also responded well to an IGF generation test. The patients were divided into two groups as good responders [height increment >1 standard deviation (SD)] and poor responders (height increment <1 SD) at the end of the follow-up period.

**Results:**

The height of the patients improved from -3.16±0.46 SD score (SDS) to -1.9±0.66 SDS. At the end of the follow-up period, mean height SDS was -1.72. Eleven of the patients showed a good response to treatment. Clinical parameters were essentially similar in the good responders and the poor responders groups. A female preponderance was noted in the good responders group.

**Conclusion:**

rGH treatment can safely be used in ISS children. Long-term GH treatment will ameliorate the height deficit and almost 40% of patients may reach their target height.

**WHAT IS ALREADY KNOWN ON THIS TOPIC?**

Recombinant growth hormone (rGH) treatment has already been used in idiopathic short stature (ISS) children without GH deficiency. Insulin-like growth factor-1 (IGF-1) deficiency accompanied some portion of the cases.

**WHAT THIS STUDY ADDS?**

Little information exists about the final height of ISS children with low IGF-1 receiving rGH treatment. In this study, we investigated and presented our findings regarding the response to rGH and the final height in a group of ISS children with low IGF-1 level.

## INTRODUCTION

An individual with idiopathic short stature (ISS) is defined as one with a height of more than 2 or 2.25 standard deviations (SDs) below the mean height for a particular age and sex, without any evidence of an underlying disorder (1,2,3). Approximately 80% of individuals with short stature do not have an identified etiology and are therefore classified as ISS. Actually, ISS consists of a heterogeneous group of individuals with short stature, and a number of them may have some elements of growth hormone (GH)/insulin-like growth factor-1 (IGF-1) axis abnormalities (4). Disorders of GH/IGF-1 axis are the most important factors affecting postnatal growth. While GH is the major regulator of circulating IGF-1 levels in healthy subjects, low IGF-1 levels can be seen with normal or high GH secretion and these cases are identified as IGF-1-deficient patients (5).

Although they show no evidence of endocrine or other causes of short stature, a small group of ISS patients have varying degrees of IGF-1 deficiency. The degree of IGF-1 deficiency in these children is usually less marked than that in severe GH deficiency or in classical severe primary IGF-1 deficiency (Laron syndrome) patients (6) These non-GH-deficient (non-GHD) short stature children with low IGF-1 also cannot attain a normal adult height (3).

In ISS children, the most effective treatment modalities for short stature are still under evaluation. rGH or rIGF-1 can be assumed to be an alternative in the treatment of these children. Recombinant human IGF-1 (rhIGF-1) treatment has been approved in only severe primary IGF-1 deficiency and had not been commercially available in most countries until very recently. Especially after the Food and Drug Administration (FDA) approval of GH for therapy in ISS in 2003, rGH has been used by different study groups (3,4,7,8,9). While short-term studies demonstrated a benefit of rGH therapy, the effect on final height (FH) and long-term results are limited and heterogeneous.

The aim of the current study was to determine the effect of rGH treatment, including long-term follow-up results, in ISS children with growth failure of unknown etiology and with IGF-1 levels below the -2 SDs of normal levels.

## METHODS

The study was approved by the Ethics Committees of the University and conducted in the Pediatric Endocrinology Clinic between 1998 and 2014. The study group consisted of children with ISS and only those with low IGF-1 levels. The diagnosis of ISS was based on the finding of a standing height of more than 2 SDs below the corresponding mean height for a particular age and sex without evidence of an underlying disorder.

Inclusion criteria were: 1) Presence of short stature (height SD below -2) and/or low growth velocity (<25th centile over 6 months of follow-up) for age and sex; 2) Adequate GH response to GH stimulation tests (peak GH >10 ng/mL); 3) Low serum IGF-1 levels (<3th centile for age and sex); 4) Adequate IGF-1 response to IGF-1 generation tests (an increase of IGF-1 higher than 15 micrograms/L in the IGF generation test; and an increase of IGFBP-3 higher than 0.4 mg/L); and 5) Receiving recombinant human GH therapy (10). Exclusion criteria were: (1) patients with identified causes of short stature (skeletal dysplasia, chronic diseases, chromosomal abnormalities, nutritional disorders) and those on any medication other than GH, such as steroid therapy, and (2) children showing a normal velocity in growth.

Clinical characteristics including birth weight, height, weight, height SDS (HSDS), body mass index (BMI), and bone age at diagnosis were determined. SDS values for anthropometric data and BMI (weight(kg)/height(m)2) were calculated using national data (11,12). Treatment duration, height increment, and FH of patients were also evaluated. FH was defined as a state in which a height velocity (HtV) less than 0.5 cm/yr had been attained (13).

Parent-adjusted height deficit was determined in all patients by calculation of differences between HSDS for chronological age and target HSDS (14).

Laboratory assessment included measurement of hematological, biochemical, and hormonal [thyroxine (T4), thyroid-stimulating hormone (TSH), IGF-I, IGF-binding protein 3 (IGF-BP3), GH stimulation tests] parameters. Serum GH (ng/mL) levels were measured by immunoradiometric assay (IRMA) using an Immunotech® kit. IGF-I and IGFBP3 were assayed by IRMA using a DSL® kit.

A diagnosis of GHD was excluded by the findings of peak stimulated GH levels higher than 10 ng/mL in the insulin tolerance test and the L-dopa stimulation test. Blood samples were drawn from an indwelling catheter inserted in an antecubital vein. All tests were performed in the morning between 08:00-09:00 am after the patients had fasted overnight.

An IGF generation test was applied to patients with low IGF-1 levels who showed a low growth velocity (by age and sex), but who had a normal or high GH response to the GH stimulation test. The IGF-1 generation test was conducted by administering rGH in a dose of 0.033 mg/kg/day for four days (10).

In patients who showed an adequate GH response to the IGF generation test, rGH therapy was given in a dose of 0.2 mg/kg/week. Improvement in height was assessed as delta HSDS (difference between HSDS at the start of the GH therapy and last HSDS). The duration of GH treatment, achieved FH, and parent-adjusted height were evaluated in all patients. According to height improvement, the patients were divided into two groups as good responders to GH treatment (∆HSDS >1 SD) and poor responders to GH treatment (∆HSDS <1 SD).

GH treatment was discontinued after epiphyseal closure, as determined by bone age evaluation.

### Statistical Analysis

In addition to descriptive analysis, t-test and Mann-Whitney U test were applied in the evaluation variables after checking the appropriateness for normal distribution. Categorical variables were compared using the chi-square test.

## RESULTS

During the 16 years of study period, 21 patients (10 girls, 11 boys) were evaluated. At admission, the mean age of the patients was 11.02±2.01 years and their mean HSDS was -3.16±0.46 before GH treatment. Mean growth velocity was 2.83±0.56 cm/year (Table 1). All patients had low IGF-1 levels and peak GH response to GH stimulation test was 17.93±8.21 ng/mL. Patients were given 0.2 mg/kg/week rGH for 3.62±0.92 years. After cessation of GH therapy, they were followed-up for 1.8 years. Total follow-up period of patients was 5.42±1.67 years. During this time, mean bone age increased from 8.38±2.25 years to 16.2±2.3 years.

The mean age of onset of puberty was 12.34±0.94 years. Girls and boys did not differ in age of onset of puberty (p=0.2). At onset of puberty, the mean HSDS was -2.77±0.61 SDS. There were no subjects with delayed puberty among the patients included in the study.

After the period of rGH treatment, the patients were evaluated with respect to their height increment. At the end of the therapy, mean HSDS was -1.9±0.66. At the last examination, the total height increment was found to be 1.4±0.1.19 SD (Table 1). Parent-adjusted height deficit was -2.35±0.35 SD at the beginning of GH therapy, and there were no differences between the good responder and poor responder groups. When patients reached their FH, the poor responders showed a greater parent-adjusted height deficit than the good responders (Table 2).

**Figure 1:**
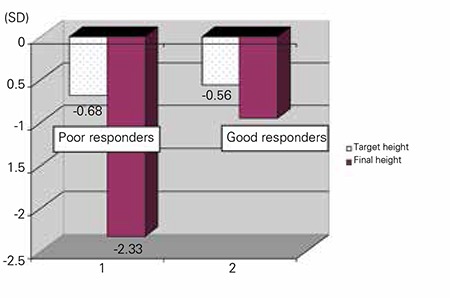
Target height and final height in the poor responder and good responder groups

When the patients were evaluated for attainment of their target height (or according to their parent-adjusted height deficit), 8 patients were found to have reached an adult height within ±1 SD of their target height. Only one patient showed a FH SDS greater than +1 SDS from his target HSDS. FH SDS values were lower than 1 SD of target HSDS in 12 patients (Figure 1).

Height improvement was greater than 1 SD in 11 patients (good responders). The remaining 10 patients showed a height increment less than 1 SD (poor responder). There were no statistical differences between the groups of good responders and poor responders with respect to birth weight, age of diagnosis, bone age at diagnosis, target height, peak GH in response to GH stimulation, increment in IGF-1 levels to IGF generation test, duration of GH treatment, age of onset of puberty, HSDS at onset of puberty, and age of GH cessation. Female preponderance was observed among the good responders (73% vs. 30%) (Table 2). Only two patients were small for gestational age, one each in the good responders and the poor responders groups.

Age of onset of puberty was similar in the poor responders (12.58±1.09 years) and good responders (12.09±0.77 years) groups (p=0.17). Also, HSDS at onset of puberty was not statistically different between the two groups. Mean HSDS of the poor responders was -2.61±0.64, while that of the good responders group was -3.13±0.44 (p=0.05).

At the cessation of GH therapy, the HSDS of the good responders group was -1.65±1.47, while this value was -2.44±0.46 SDS in the poor responders.

When defining the parent-adjusted height deficit as lower than 1 SDS, only two patients in the poor responders group were able to reach an acceptable target HSDS.

At the onset of the study, the mean BMI SDS of the study group was -1.11±1.09. At the end of the follow-up, it increased to 0.11±0.88 (p=0.0002). While there was no differences between the BMI SDS values of the poor responders and those of the good responders initially, good responders showed a greater increase in BMI SDS after treatment than poor responders (Table 1, 2).

## DISCUSSION

The underlying abnormalities in ISS children may be associated with abnormalities affecting the integrity of the GH receptor or signal transduction pathway, a state which may have compromised the sensitivity to GH (4). There may be a spectrum of GH insensitivity ranging from mild, as in some ISS patients, to severe, as in the Laron syndrome (4). In rare cases, ISS is due to molecular abnormalities of IGF. Some researchers suggest that an overlap exists between ISS patients and those with partial or atypical GH insensitivity (15). Of all ISS patients, IGF-1 deficient patients constitute a very small group and they may have some degree of GH insensitivity. Excluding the severe IGF deficiency cases, the prevalence of mild IGF deficiency in ISS children is not well-known. In a French cohort study, the prevalence of severe primary IGF deficiency was reported as 2.5% among the ISS children (7). In another study of prepubertal children with isolated short stature below or equal to -2.5 SDS, the prevalence of IGF deficiency has been reported as 0.8% (16).

We evaluated a selected subgroup of IGF-1 deficient ISS children who showed an adequate response to a GH stimulation test and also an adequate response to a IGF-1 generation test, indicating that they could benefit from rGH treatment. Although there are reports on GH treatment in ISS children, the definition of short stature (below -2, -2.25,-2.5,-3 SDS), of IGF-1 levels (normal, -2 SDS, -3 SDS), the doses of GH, etc. were heterogeneous in these studies. Overall, these studies reported good responses to GH in these children (7,8,9,16,17,18).

In our patients, after treatment with GH for a mean period of 3.62 years, HSDS increased from -3.16 SD to -1.9 SD. Treatment with rGH was well-tolerated. It did not significantly accelerate bone maturation and no overall side effects were seen. To date, rGH treatment in GHD as well as in non-GHD children has shown a good safety and efficiency profile (19).

While short-term studies showed an approximately +0.7 SD HSDS increment over one year, adult height studies suggested a height gain of 0.57 SD in ISS children treated with GH (20). However, the studies on ISS are quite heterogeneous. Many of these studies have been conducted on groups including both IGF-1-deficient ISS children and short children with normal IGF-1 levels. However, although the doses used and the characteristics of patients were not similar in all these studies, FH of ISS children treated with GH was reported to be 0.6 SDS and 0.5 SDS greater than the control groups (15,19). A meta-analysis consisting of reports on controlled studies up to FH in children with ISS showed a mean height difference of 0.65 SDS from that of the controls (18).

ISS patients do not show a unique response to GH treatment. Even in GHD patients, there was no uniform response to GH. The sensitivity of individual patients to treatment can be different. Some ISS patients showed a good response, unlike others who did not respond well (4,21). We grouped our patients as good responders and poor responders according to their height improvement. We attempted to rule out poor compliance with scheduled GH injections by asking the families to return empty used cartridges and asking about any misinjection day at every visit. There were no statistical differences between the two groups with respect to birth weight, chronological age at presentation, HSDS at admission, bone age at diagnosis, target height, peak GH to GH stimulation test, increment in IGF-1 levels in response to the IGF generation test, duration of GH treatment, age of onset of puberty, HSDS at puberty onset, and age when GH treatment was stopped. However, a preponderance of girls was noted in the good responders group, as opposed to that in the poor responders group.

Although the parameters for predicting long-term response to GH in ISS have not been established, some studies showed that greater height gain was associated with a lower baseline HSDS, a lower baseline IGF-1 level, a lower pretreatment HtV, and with a greater delay in bone age (15). In fact, the characteristics of etiological factors leading to ISS could be predictive for response to GH therapy. In ISS patients with a defect of GHR, the response can be lower than that of patients with a disorganized pattern of GH release (22).

ISS can be accompanied by familial short stature and/or constitutional delay of growth and puberty (CGDP) in some patients (23). Some authors have suggested that children with ISS who responded more vigorously to rGH probably have CGDP (23). In our cases, puberty was not delayed in both good responder and poor responder groups. So, the effect of CGPD does not seem to be a component of short stature in these patients.

Groups were not equal according to sex. The rate of female patients was higher in good responders than in poor responders. In other words, we can say that poor response was more frequent in male patients. We could not explain this difference between female and male patients. However, this result may not be conclusive and may have been related to the relative smallness of our sample.

The familial components of short stature were also evaluated in our patients. Mean target HSDS was -0.62 and there was no difference between the two groups. Only one patient had a target height lower than -2 SD, and this patient belonged to the poor responders group. Except for this patient, it could not be said that an additional familial component of short stature was found in this study.

At FH evaluation, 9 patients (43%) were found to have reached or exceeded their target height. As expected, these patients were mostly in the good responders group. It can be suggested that an important part of slowly growing ISS patients with low IGF-1 levels may show a good response to rGH therapy.

We used a fixed dose of 0.2 mg/kg/week of GH in our patients. An IGF-1 based GH dosing could be an alternative treatment in patients in the poor responders group. In one study on non-GHD subjects with significant short stature and different IGF-I levels, adjusting the GH dose to achieve an IGF-I level in the mid-to-upper normal range has resulted in a significant increase in HSDS at 12 months and has been well-tolerated (3). The authors have concluded that this approach used mean GH doses within FDA-approved ranges for patients with ISS and allowed for optimized height gain, compared with standard fixed doses of GH in children with ISS.

One other aim of our study was to elucidate the possibilities of our patients having monogenic causes of short stature. Unfortunately, we had no possibility to make molecular genetic analysis in our patients. Monogenic causes of short stature which are associated with a low serum IGF-I level and normal or high GH levels could comprise the defects of GH1 (bioinactive GH), GHSR (ghrelin receptor), GHR (GH receptor), STAT5B, IGF1, and IGFALS (24). In one study, heterozygous STAT5B mutations, with or without heterozygous IGFALS defects were shown to be present in short children with GH insensitivity. In the same study, functional variants were found in children with less severe short stature or IGF-I deficiency (24). In the future, with advancing molecular genetic studies, it will be possible to reveal the underlined etiological factors of ISS patients.

In conclusion, our study shows that slowly growing ISS children with IGF deficiency can respond well to rGH treatment. Knowing underlying molecular abnormalities in ISS patients could be helpful for planning the treatment in many, if not all, cases. Especially if there is no opportunity to make advanced molecular genetic analysis in non-GHD short children with IGF deficiency, rGH treatment can be beneficial. In this situation, long-term GH treatment will ameliorate height deficit, and almost 40% of patients will be able to reach their target height.

## Figures and Tables

**Table 1:**
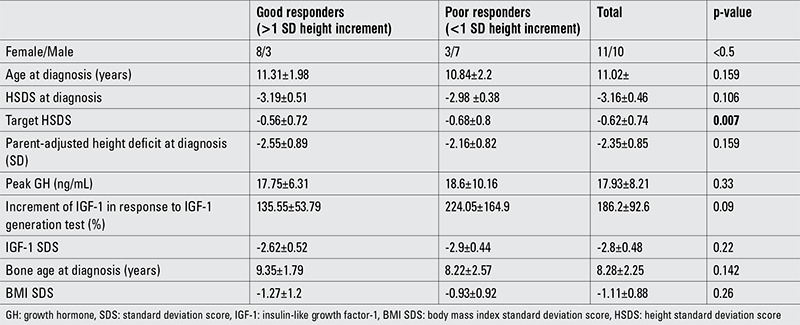
Characteristics of patients with good and poor response to growth hormone treatment

**Table 2:**
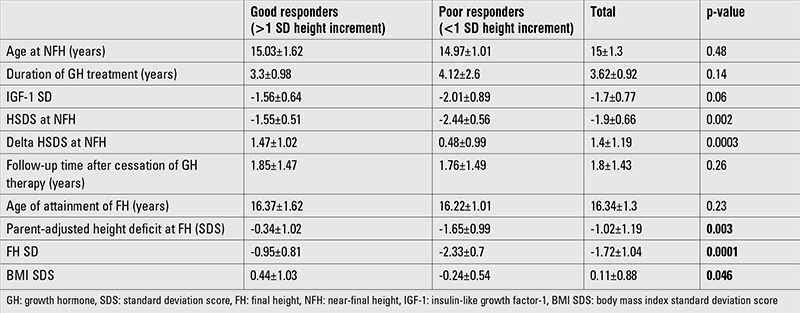
Characteristics of patients with good and poor response to growth hormone treatment
